# Crystal structure of isopropyl 2-hy­droxy-2-phenyl­acetate: a pharmacopoeia reference standard

**DOI:** 10.1107/S2056989017005862

**Published:** 2017-04-28

**Authors:** Ivan Isaiev, Svitlana Shishkina, Igor Ukrainets, Elena Bevz

**Affiliations:** a V. N. Karazin Kharkiv National University, 4 Svobody Sq., Kharkiv 61077, Ukraine; b SSI ‘Institute for Single Crystals’, National Academy of Sciences of Ukraine, 60 Nauky Ave., Kharkiv 61001, Ukraine; c National University of Pharmacy, 4 Valentinivska St., Kharkiv 61168, Ukraine

**Keywords:** crystal structure, pharmacopoeia reference standard, hydrogen bonding.

## Abstract

The title compound, C_11_H_14_O_3_, is used as a pharmacopoeia reference standard for determining impurities in the drug Pregabalin, used for the treatment of epilepsy and diabetic neuropathic pain. The mol­ecule is far from being planar, with the dihedral angle between the planes of the aromatic ring and the carboxyl fragment (O—C=O) being 76.1 (6)°. The isopropyl substituent is located in a synperiplanar position relative to the C=O bond and is turned so that the C—O—C—H torsion angle is −43.7°. In the crystal, bifurcated O—H⋯(O,O) hydrogen bonds, enclosing *R*_1_^2^(5) ring motifs, lead to the formation of chains propagating along the *c*-axis direction. Inversion-related chains are linked by the C—H⋯O hydrogen bonds, forming undulating layers lying parallel to the *bc* plane.

## Abstract 2

The title compound is a pharmacopoeia reference standard for determining impurities in the drug Pregabalin, used for the treatment of epilepsy and diabetic neuropathic pain.

## Chemical context   

Pharmacopoeia reference standards are used widely for identification and qu­anti­tative determination of an active ingredient and undesirable impurity contents in many drug substances (European Pharmacopoeia Supplement, 2017 ▸). The title compound is used as the pharmacopoeia reference standard for the determining the level of impurities in Pregabalin (European Pharmacopoeia Supplement, 2016 ▸). This drug, sold under the trade mark ‘Lyrica’ (Silverman, 2016 ▸) is used for the treatment of epilepsy and diabetic neuropathic pains. Until now, its mol­ecular and crystal structure were unknown.
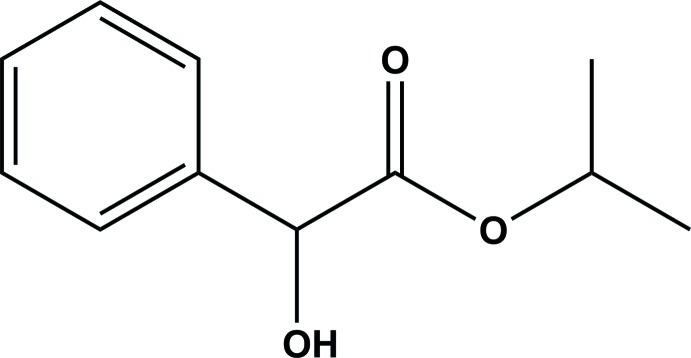


## Structural commentary   

**Figure 1:**
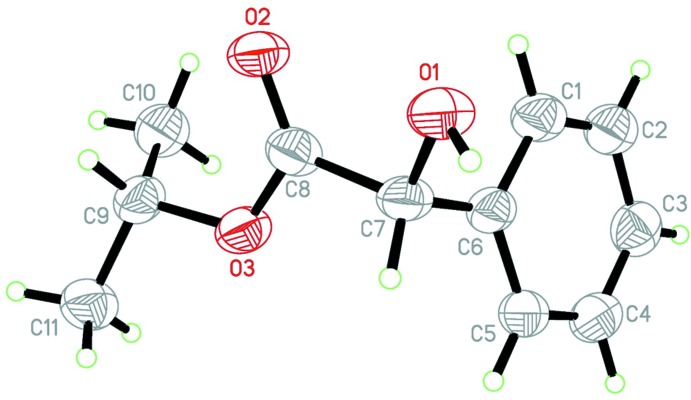
The mol­ecular structure of the title compound, showing the atom labelling. Displacement ellipsoids are drawn at the 50% probability level.

The mol­ecular structure of the title compound is shown in Fig. 1 ▸. The hydroxyl group is situated in the -*sc* position relative to the C1—C6 endocyclic bond; torsion angle C1—C6—C7—O1 being −46.2 (6)°). The ester substituent at atom C7 has a +*sc*-orientation with respect to bond C1—C6 bond, with torsion angle C1—C6—C7—C8 = 71.2 (6)°, and it is turned in such way that the dihedral angle between the planes of the aromatic ring (C1–C6) and the carboxyl fragment (O3—C8=O2) is 76.1 (6)°. The isopropyl substituent is located in a *syn*-periplanar position relative to the C8=O2 bond and is turned so that the C8—O3—C9—H9 torsion angle is −43.7°.

## Supra­molecular features   

**Figure 2:**
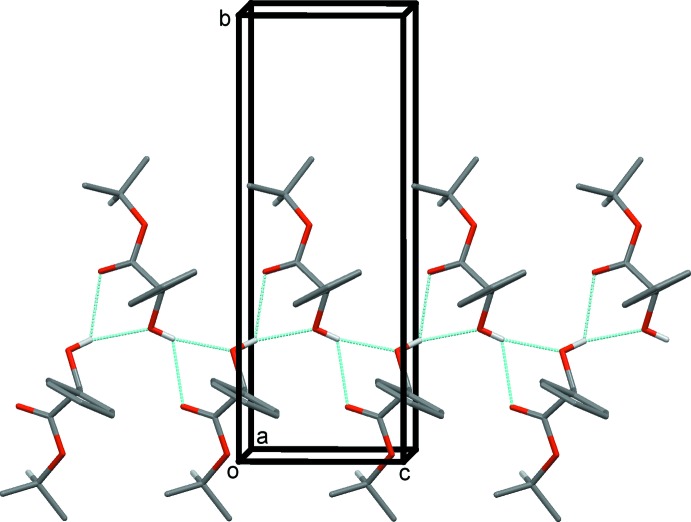
A partial view along the *a* axis of the crystal packing of the title compound, with the hydrogen bonds shown as dashed lines (see Table 1 ▸). For clarity, only H atoms H1*O* and H9 have been included.

**Figure 3:**
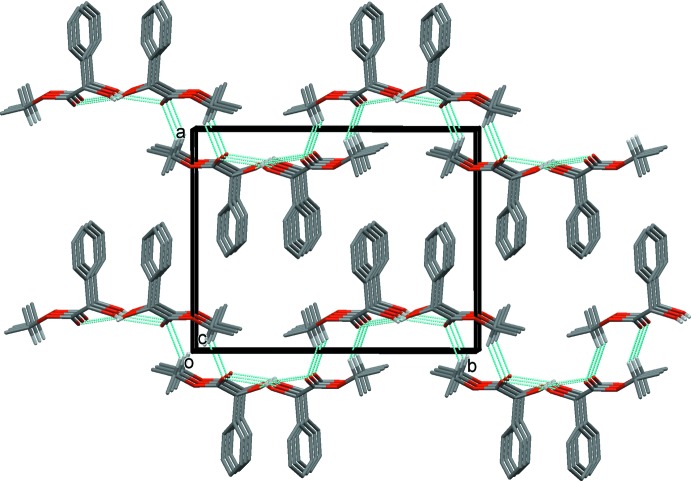
A view along the *c* axis of the crystal packing of the title compound, with the hydrogen bonds shown as dashed lines (see Table 1 ▸). For clarity, only H atoms H1*O* and H9 have been included.

In the crystal, mol­ecules are linked by bifurcated O—H⋯(O,O) hydrogen bonds, forming chains propagating along [001] and enclosing 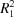
(5) ring motifs (Fig. 2 ▸ and Table 1 ▸). Neighbouring chains are linked by C—H⋯O hydrogen bonds, forming undulating layers lying parallel to the *bc* plane (Table 1 ▸ and Fig. 3 ▸).

## Database survey   

A search in the Cambridge Structural Database (Version 5.38, update February 2017; Groom *et al.*, 2016 ▸) for substructure isopropyl 2-hy­droxy-2-phenyl­acetate yielded three hits, *viz.* isopropyl 2,2-bis­(4-bromo­phen­yl)-2-hy­droxy­acetate (EFAFEY; Smith, 2012 ▸), 1-isopropyl 4-methyl 2-hy­droxy-2-{2-[(meth­oxy­carbon­yl)amino]­phen­yl} succinate (MAZJAA; Suárez-Castillo *et al.*, 2012 ▸) and *syn*-isopropyl 2,3-dihy­droxy-4-methyl-2-phenyl­penta­noate (MERRIL; Scholtis *et al.*, 2006 ▸). In the crystals of these three compounds, mol­ecules are linked by pairs of O—H⋯O hydrogen bonds, forming inversion dimers.

## Synthesis and crystallization   

**Figure 4:**
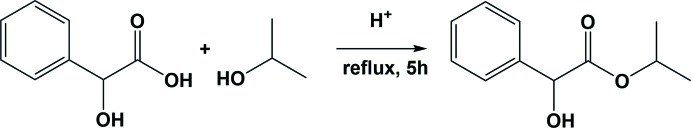
Reaction scheme

To a solution of (2*RS*)-2-hy­droxy-2-phenyl­acetic acid (15.22 g, 0.1 mol; racemic mandelic acid) in 50 ml propan-2-ol was added 0.5 ml of concentrated H_2_SO_4_, and the mixture was refluxed for 5 h (Fig. 4 ▸). The excess of propan-2-ol was removed *in vacuo*. The reaction mixture was diluted with cold water and Na_2_CO_3_ was added to adjust the pH to 8. The solution was extract with CH_2_Cl_2_ (3 × 30 ml). The organic layers were combined and the solvent extracted by distillation (at reduced pressure at the end). The residue was distilled *in vacuo*, and a fraction with a boiling point of 361–363 K/4 mm Hg was taken, and then left for several hours in the refrigerator at *ca* 278 K, giving finally the title compound as colourless needle-like crystals (yield of 17.67 g, 91%; m.p. 306.9–307.3 K).

## Refinement   

Crystal data, data collection and structure refinement details are summarized in Table 2 ▸. All of the H atoms could all be located from difference-Fourier maps. The hydroxyl H atom was refined with *U*_iso_(H) = 1.5*U*_eq_(O). The C-bound H atoms were included in calculated positions and treated as riding: C—H = 0.93–0.97 Å, with *U*_iso_(H) = 1.5*U*_eq_(C-meth­yl) and 1.2*U*_eq_(C) for other H atoms.

## Supplementary Material

Crystal structure: contains datablock(s) I, Global. DOI: 10.1107/S2056989017005862/su5365sup1.cif

Structure factors: contains datablock(s) I. DOI: 10.1107/S2056989017005862/su5365Isup2.hkl

Supporting information file. DOI: 10.1107/S2056989017005862/su5365Isup3.cml

CCDC reference: 1544853

Additional supporting information:  crystallographic information; 3D view; checkCIF report

## Figures and Tables

**Table 1:** Hydrogen-bond geometry (Å, °)

*D*—H⋯*A*	*D*—H	H⋯*A*	*D*⋯*A*	*D*—H⋯*A*
O1—H1*O*⋯O1^i^	0.83 (6)	2.12 (5)	2.903 (2)	158 (5)
O1—H1*O*⋯O2^i^	0.83 (6)	2.38 (6)	2.930 (5)	124 (5)
C9—H9⋯O2^ii^	1.00	2.53	3.379 (7)	142

Symmetry codes: (i) 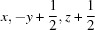; (ii) 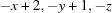.

**Table 2:** Experimental details

Crystal data	
Chemical formula	C_11_H_14_O_3_
*M*_r_	194.22
Crystal system, space group	Monoclinic, *P*2_1_/*c*
Temperature (K)	100
*a*, *b*, *c* (Å)	11.872 (3), 15.165 (4), 5.6079 (11)
β (°)	91.41 (2)
*V* (Å^3^)	1009.3 (4)
*Z*	4
Radiation type	Mo *K*α
μ (mm^−1^)	0.09
Crystal size (mm)	0.20 × 0.08 × 0.06

Data collection	
Diffractometer	Agilent Xcalibur Sapphire3
Absorption correction	Multi-scan (*CrysAlis RED*; Agilent, 2012 ▸)
*T*_min_, *T*_max_	0.357, 1.000
No. of measured, independent and observed [*I* > 2σ(*I*)] reflections	5292, 1761, 922
*R*_int_	0.101
(sin θ/λ)_max_ (Å^−1^)	0.594

Refinement	
*R*[*F* ^2^ > 2σ(*F* ^2^)], *wR*(*F* ^2^), *S*	0.090, 0.255, 1.03
No. of reflections	1761
No. of parameters	132
H-atom treatment	H atoms treated by a mixture of independent and constrained refinement
Δρ_max_, Δρ_min_ (e Å^−3^)	0.30, −0.26

Computer programs: *CrysAlis CCD* and *CrysAlis RED* (Agilent, 2012 ▸), *SHELXS2014* (Sheldrick, 2008 ▸), *SHELXL2014* (Sheldrick, 2015 ▸), *ORTEP-3 for Windows* (Farrugia, 2012 ▸), *Mercury* (Macrae *et al.*, 2008 ▸), *PLATON* (Spek, 2009 ▸) and *publCIF* (Westrip, 2010 ▸).

## supplementary crystallographic information

### Crystal data

**Table:**

C_11_H_14_O_3_	*F*(000) = 416
*M**_r_* = 194.22	*D*_x_ = 1.278 Mg m^−^^3^
Monoclinic, *P*2_1_/*c*	Mo *K*α radiation, λ = 0.71073 Å
*a* = 11.872 (3) Å	Cell parameters from 403 reflections
*b* = 15.165 (4) Å	θ = 4.3–21.1°
*c* = 5.6079 (11) Å	µ = 0.09 mm^−^^1^
β = 91.41 (2)°	*T* = 100 K
*V* = 1009.3 (4) Å^3^	Needle, colourless
*Z* = 4	0.20 × 0.08 × 0.06 mm

### Data collection

**Table:**

Agilent Xcalibur Sapphire3 diffractometer	1761 independent reflections
Radiation source: Enhance (Mo) X-ray Source	922 reflections with *I* > 2σ(*I*)
Detector resolution: 16.1827 pixels mm^-1^	*R*_int_ = 0.101
ω–scan	θ_max_ = 25.0°, θ_min_ = 3.2°
Absorption correction: multi-scan (CrysAlis RED; Agilent, 2012)	*h* = −14→13
*T*_min_ = 0.357, *T*_max_ = 1.000	*k* = −17→18
5292 measured reflections	*l* = −5→6

### Refinement

**Table:**

Refinement on *F*^2^	Primary atom site location: structure-invariant direct methods
Least-squares matrix: full	Secondary atom site location: difference Fourier map
*R*[*F*^2^ > 2σ(*F*^2^)] = 0.090	Hydrogen site location: difference Fourier map
*wR*(*F*^2^) = 0.255	H atoms treated by a mixture of independent and constrained refinement
*S* = 1.03	*w* = 1/[σ^2^(*F*_o_^2^) + (0.1022*P*)^2^] where *P* = (*F*_o_^2^ + 2*F*_c_^2^)/3
1761 reflections	(Δ/σ)_max_ < 0.001
132 parameters	Δρ_max_ = 0.30 e Å^−^^3^
0 restraints	Δρ_min_ = −0.26 e Å^−^^3^

### Special details

**Table:**

Geometry. All esds (except the esd in the dihedral angle between two l.s. planes) are estimated using the full covariance matrix. The cell esds are taken into account individually in the estimation of esds in distances, angles and torsion angles; correlations between esds in cell parameters are only used when they are defined by crystal symmetry. An approximate (isotropic) treatment of cell esds is used for estimating esds involving l.s. planes.

### Fractional atomic coordinates and isotropic or equivalent isotropic displacement parameters (Å^2^)

**Table:**

	*x*	*y*	*z*	*U*_iso_*/*U*_eq_	
O1	0.8289 (3)	0.2748 (2)	0.4019 (6)	0.0456 (10)	
H1O	0.845 (4)	0.252 (4)	0.534 (10)	0.068*	
O2	0.8740 (3)	0.4013 (2)	0.0966 (6)	0.0476 (11)	
O3	0.8375 (3)	0.5068 (2)	0.3667 (6)	0.0455 (10)	
C1	0.5984 (5)	0.3361 (3)	0.3003 (9)	0.0443 (14)	
H1	0.6314	0.3108	0.1638	0.053*	
C2	0.4827 (5)	0.3407 (3)	0.3113 (9)	0.0479 (14)	
H2	0.4368	0.3171	0.1856	0.057*	
C3	0.4332 (5)	0.3801 (3)	0.5079 (9)	0.0492 (15)	
H3	0.3535	0.3852	0.5142	0.059*	
C4	0.4998 (5)	0.4111 (3)	0.6910 (9)	0.0467 (14)	
H4	0.4658	0.4367	0.8262	0.056*	
C5	0.6154 (5)	0.4061 (3)	0.6831 (9)	0.0419 (13)	
H5	0.6600	0.4286	0.8122	0.050*	
C6	0.6684 (4)	0.3679 (3)	0.4857 (8)	0.0387 (13)	
C7	0.7937 (4)	0.3603 (3)	0.4744 (8)	0.0403 (13)	
H7	0.8283	0.3743	0.6343	0.048*	
C8	0.8399 (4)	0.4230 (3)	0.2895 (9)	0.0416 (13)	
C9	0.8869 (5)	0.5744 (3)	0.2113 (9)	0.0452 (14)	
H9	0.9599	0.5521	0.1494	0.054*	
C10	0.8087 (5)	0.5957 (4)	0.0050 (9)	0.0565 (16)	
H10A	0.7346	0.6120	0.0649	0.085*	
H10B	0.8009	0.5440	−0.0989	0.085*	
H10C	0.8394	0.6451	−0.0856	0.085*	
C11	0.9094 (5)	0.6527 (3)	0.3724 (9)	0.0528 (15)	
H11A	0.9489	0.6329	0.5183	0.079*	
H11B	0.8378	0.6802	0.4136	0.079*	
H11C	0.9562	0.6956	0.2897	0.079*	

### Atomic displacement parameters (Å^2^)

**Table:**

	*U*^11^	*U*^22^	*U*^33^	*U*^12^	*U*^13^	*U*^23^
O1	0.074 (3)	0.030 (2)	0.0327 (19)	0.0060 (18)	0.0021 (18)	0.0017 (15)
O2	0.074 (3)	0.040 (2)	0.0298 (19)	−0.0036 (18)	0.0078 (18)	−0.0039 (15)
O3	0.068 (3)	0.035 (2)	0.0337 (19)	−0.0031 (18)	0.0107 (17)	−0.0007 (15)
C1	0.062 (4)	0.039 (3)	0.031 (3)	0.000 (3)	−0.003 (2)	0.003 (2)
C2	0.064 (4)	0.042 (3)	0.038 (3)	−0.001 (3)	−0.003 (3)	0.005 (2)
C3	0.055 (4)	0.046 (3)	0.047 (3)	−0.001 (3)	−0.001 (3)	0.009 (3)
C4	0.063 (4)	0.036 (3)	0.041 (3)	0.004 (3)	0.010 (3)	0.001 (2)
C5	0.065 (4)	0.027 (3)	0.033 (3)	−0.004 (3)	0.001 (2)	0.003 (2)
C6	0.055 (4)	0.033 (3)	0.029 (3)	−0.004 (2)	0.003 (2)	0.003 (2)
C7	0.060 (4)	0.031 (3)	0.030 (3)	−0.001 (3)	0.003 (2)	−0.004 (2)
C8	0.055 (4)	0.034 (3)	0.036 (3)	−0.002 (3)	−0.003 (2)	−0.002 (2)
C9	0.061 (4)	0.038 (3)	0.037 (3)	−0.006 (3)	0.012 (2)	0.001 (2)
C10	0.081 (4)	0.051 (4)	0.038 (3)	−0.015 (3)	0.001 (3)	0.010 (3)
C11	0.074 (4)	0.039 (3)	0.045 (3)	−0.008 (3)	0.002 (3)	−0.001 (2)

### Geometric parameters (Å, º)

**Table:**

O1—C7	1.424 (6)	C3—C4	1.364 (8)
O2—C8	1.211 (5)	C4—C5	1.376 (8)
O3—C8	1.343 (6)	C5—C6	1.412 (7)
O3—C9	1.476 (6)	C6—C7	1.495 (7)
C1—C2	1.378 (7)	C7—C8	1.519 (7)
C1—C6	1.401 (7)	C9—C10	1.501 (8)
C2—C3	1.397 (7)	C9—C11	1.512 (7)
			
C8—O3—C9	117.1 (4)	O1—C7—C6	112.4 (4)
C2—C1—C6	121.6 (5)	O1—C7—C8	105.2 (4)
C1—C2—C3	119.8 (5)	C6—C7—C8	110.9 (4)
C4—C3—C2	119.6 (6)	O2—C8—O3	123.8 (5)
C3—C4—C5	121.1 (5)	O2—C8—C7	125.0 (4)
C4—C5—C6	120.8 (5)	O3—C8—C7	111.2 (4)
C1—C6—C5	117.0 (5)	O3—C9—C10	111.0 (4)
C1—C6—C7	121.0 (5)	O3—C9—C11	105.0 (4)
C5—C6—C7	121.9 (5)	C10—C9—C11	112.9 (5)
			
C6—C1—C2—C3	1.9 (8)	C1—C6—C7—C8	71.2 (6)
C1—C2—C3—C4	−2.1 (8)	C5—C6—C7—C8	−109.5 (5)
C2—C3—C4—C5	1.4 (8)	C9—O3—C8—O2	−4.3 (8)
C3—C4—C5—C6	−0.5 (7)	C9—O3—C8—C7	176.1 (4)
C2—C1—C6—C5	−0.9 (7)	O1—C7—C8—O2	15.9 (7)
C2—C1—C6—C7	178.4 (5)	C6—C7—C8—O2	−105.9 (6)
C4—C5—C6—C1	0.2 (7)	O1—C7—C8—O3	−164.5 (4)
C4—C5—C6—C7	−179.1 (4)	C6—C7—C8—O3	73.7 (5)
C1—C6—C7—O1	−46.2 (6)	C8—O3—C9—C10	76.8 (6)
C5—C6—C7—O1	133.0 (5)	C8—O3—C9—C11	−160.9 (5)

### Hydrogen-bond geometry (Å, º)

**Table:**

*D*—H···*A*	*D*—H	H···*A*	*D*···*A*	*D*—H···*A*
O1—H1*O*···O1^i^	0.83 (6)	2.12 (5)	2.903 (2)	158 (5)
O1—H1*O*···O2^i^	0.83 (6)	2.38 (6)	2.930 (5)	124 (5)
C9—H9···O2^ii^	1.00	2.53	3.379 (7)	142

Symmetry codes: (i) *x*, −*y*+1/2, *z*+1/2; (ii) −*x*+2, −*y*+1, −*z*.

## References

[bb1] Agilent (2012). *CrysAlis PRO*. Agilent Technologies, Yarnton, England.

[bb2] European Pharmacopoeia Supplement (2016). pp. 5801–5803. Strasbourg: Council of Europe.

[bb3] European Pharmacopoeia Supplement (2017). pp. 733–736. Strasbourg: Council of Europe.

[bb4] Farrugia, L. J. (2012). *J. Appl. Cryst.* **45**, 849–854.

[bb5] Groom, C. R., Bruno, I. J., Lightfoot, M. P. & Ward, S. C. (2016). *Acta Cryst.* B**72**, 171–179. 10.1107/S2052520616003954 PMC4822653 27048719

[bb6] Macrae, C. F., Bruno, I. J., Chisholm, J. A., Edgington, P. R., McCabe, P., Pidcock, E., Rodriguez-Monge, L., Taylor, R., van de Streek, J. & Wood, P. A. (2008). *J. Appl. Cryst.* **41**, 466–470.

[bb7] Scholtis, S., Ide, A. & Mahrwald, R. (2006). *Org. Lett.* **8**, 5353–5355. 10.1021/ol062252w 17078716

[bb8] Sheldrick, G. M. (2008). *Acta Cryst.* A**64**, 112–122. 10.1107/S0108767307043930 18156677

[bb9] Sheldrick, G. M. (2015). *Acta Cryst.* C**71**, 3–8.

[bb10] Silverman, R. B. (2016). *Technol. Innov.* **17**, 153–158.

[bb11] Smith, G. (2012). *Acta Cryst.* E**68**, o3276. 10.1107/S1600536812044571 PMC3588824 23468789

[bb12] Spek, A. L. (2009). *Acta Cryst.* D**65**, 148–155. 10.1107/S090744490804362X PMC2631630 19171970

[bb13] Suárez-Castillo, R., Bautista-Hernández, C. I., Sánchez-Zavala, M., Meléndez-Rodríguez, M., Sierra-Zenteno, A., Morales-Ríos, M. S. & Joseph-Nathan, P. (2012). *Heterocycles*, **85**, 2147–2171.

[bb14] Westrip, S. P. (2010). *J. Appl. Cryst.* **43**, 920–925.

